# P-1975. Disproportionality Analysis of Antimicrobial-Induced Nephrotoxicity: Comparative Safety Signals Across Beta-Lactams, Aminoglycosides, Glycopeptides, and Polymyxins

**DOI:** 10.1093/ofid/ofaf695.2142

**Published:** 2026-01-11

**Authors:** Jose T John, Manu Mathew, Ashin Siby

**Affiliations:** Durdans Hospital, Colombo, Western Province, Sri Lanka; Durdans Hospital, Colombo, Western Province, Sri Lanka; Durdans Hospital, Colombo, Western Province, Sri Lanka

## Abstract

**Background:**

Nephrotoxicity is a well-recognized adverse effect of several antimicrobial agents, particularly those used in severe infections and critical care. While beta-lactams are generally considered renally safe, aminoglycosides, glycopeptides, and polymyxins carry well-documented renal risks. However, comparative post-marketing data quantifying nephrotoxicity across these drug classes remain limited. This study aimed to evaluate and compare the disproportionality of nephrotoxic adverse events associated with commonly used parenteral antimicrobials using the U.S. FDA Adverse Event Reporting System (FAERS).

**Figure:**
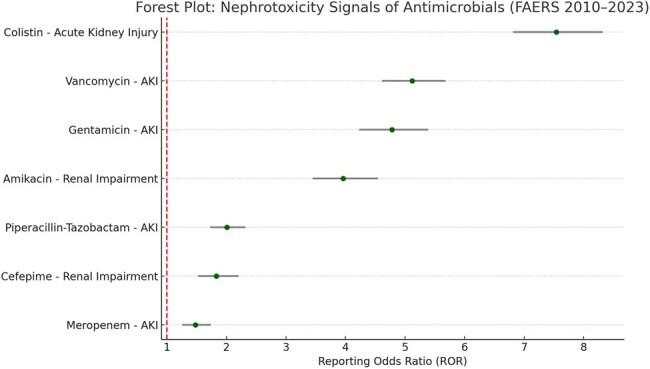
Forest Plot: Nephrotoxicity Signals of Antimicrobials (FAERS 2010–2023) This forest plot displays Reporting Odds Ratios (RORs) with 95% confidence intervals for nephrotoxicity-related adverse events associated with antimicrobial use. Colistin showed the highest signal for acute kidney injury (ROR: 7.54), followed by vancomycin (ROR: 5.12) and gentamicin (ROR: 4.78). Beta-lactams such as meropenem exhibited lower but statistically significant signals. These findings support close renal monitoring when using high-risk agents.

**Methods:**

A retrospective pharmacovigilance analysis was conducted using FAERS data from January 2010 to December 2023. Reports involving vancomycin, gentamicin, amikacin, colistin, polymyxin B, piperacillin-tazobactam, cefepime, and meropenem were extracted. Nephrotoxicity-related MedDRA Preferred Terms (PTs) included “acute kidney injury,” “renal impairment,” “interstitial nephritis,” and “renal failure.” Reporting Odds Ratios (RORs) with 95% confidence intervals (CIs) were calculated. A signal was considered significant if the ROR lower CI >1 and ≥3 cases.

**Results:**

Out of 42,786 relevant reports, the strongest signal was observed for colistin and acute kidney injury (ROR: 7.54, 95% CI: 6.81–8.32), followed by vancomycin (ROR: 5.12), and gentamicin (ROR: 4.78). Piperacillin-tazobactam and cefepime showed moderate signals (ROR: 2.01 and 1.83, respectively), while meropenem had the lowest but still significant signal (ROR: 1.48). Median time to onset was 5–7 days, with polymyxin- and glycopeptide-associated AKI more likely to result in hospitalization or dialysis.

**Conclusion:**

This FAERS-based study demonstrates a strong class-specific nephrotoxicity signal among antimicrobials, especially polymyxins, glycopeptides, and aminoglycosides. The findings emphasize the need for close renal monitoring, dose adjustments, and pharmacovigilance-based stewardship in high-risk patients receiving these agents.

**Disclosures:**

All Authors: No reported disclosures

